# Words and Slang

**Published:** 1881-04

**Authors:** J. B. Chandler


### WORDS AND SLANG.

J. B. CHANDLER.

The charge is brought, and justly, that the American people are much given to slang, that new words are constantly engrafted upon the language emanating from sources far below the accepted authorities and yet obtaining a foothold not easily shaken.

Nor is the coinage of new words entirely confined to this country or to the lower classes. We find slang phrases peculiar to fashionable society, such as "Chic," a very expressive term to the initiated; again, to do something is either "the thing," or not "the thing;" such a bonnet is "all the go," in place of fashionable. Mrs. B. "receives" on Thursday; travelers "do" Europe. Names adopted by popular novelists for prominent characters soon become synonyms for the weakness or vice portrayed, as "Oily Gammon," Uriah Heap, &c.

Many of these expressions "smell of the shop" as "there is no discount on him," "he is par funds," or "open her out wide," "clear the track." To the mining districts of the west we are indebted for large additions to the stock of slang. A business does not "pan out" well; or he has reached "hard pan;" a young man is "no account" he "has no dust in him." These are but a few of the instances of slang floating on the level from which I write; above and below are many peculiar to those levels, some of which will eventually be dignified with places in authoritative dictionaries. Not slang dictionaries of which there are several in print, but the Webster's and Worcester's of the future. I have not referred to the slang of particular tribes of human beings isolated by their habits from general society such as gipsies, tramps, thieves, &c., because theirs seems to be a special language not likely to be generally adopted, but used as a convenient cover to crime—or as a means of special recognition.

Many condemn the use of these innovations on our language; they certainly do not add to the beauty of conversation and frequently are open to the just censure of lack of refinement, yet, beginning at the lowest tiers of society we find them gradually obtaining a foothold and finally many of them become fairly engrafted on the language.

Let us consider then, if out of such material the natural growth of language is not to come, and let us not despise the soil from which it springs. Nature teaches us a lesson on this when we contemplate the sources of the best fruits of the earth.

Glancing at the roots of many words in our dictionaries, we find that time has given them far different meanings from what their sources seem to warrant.

Let us take the word "sincere," in very general use and its meaning as now accepted not the least ambiguous. It is derived from two latin words *sine* without, and *cera*, wax. Is there any great difference between speaking of a young lady you esteem as being "without wax," and a foolish young man as having "no dust in him?" In the first instance the honest truthfulness of character is represented by pure honey, in the latter the worthlessness by washings yielding no gold.

Many instances of similar character could be given, but the object is obtained by arresting the attention of the readers of the BISTOURY to the changes of language in progress and to the gradual development of slang into accepted terms.

The public press in our large cities is an important factor in these changes; brevity is not only the soul of wit, but is also a most valuable possession of the local reporter in an overcrowded paper, hence he uses words to save sentences and frequently those words although very generally understood have not before appeared in print, except in the works of authors portraying border and low life. This is the first step and soon the infant is developed into a full fledged accepted noun, adjective or verb and comes into constant use. There are instances of this kind in manufacturing verbs from nouns. One occurs not yet generally adopted but frequently used, thus—John Brown *suicided*, instead of committed suicide.

Perhaps some of the readers of the BISTOURY may find pleasure in carrying out this investigation further, and watching the gradual growth of despised slang into accepted language.

